# ABA‐inducible *DEEPER ROOTING* *1* improves adaptation of maize to water deficiency

**DOI:** 10.1111/pbi.13889

**Published:** 2022-07-22

**Authors:** Xuanjun Feng, Li Jia, Yunting Cai, Huarui Guan, Dan Zheng, Weixiao Zhang, Hao Xiong, Hanmei Zhou, Ying Wen, Yue Hu, Xuemei Zhang, Qingjun Wang, Fengkai Wu, Jie Xu, Yanli Lu

**Affiliations:** ^1^ State Key Laboratory of Crop Gene Exploration and Utilization in Southwest China Wenjing China; ^2^ Maize Research Institute, Sichuan Agricultural University Wenjing China

**Keywords:** ABA‐inducible promoter, drought, teosinte, root architecture

## Abstract

Root architecture remodelling is critical for forage moisture in water‐limited soil. *DEEPER ROOTING 1* (*DRO1*) in *Oryza*, *Arabidopsis*, and *Prunus* has been reported to improve drought avoidance by promoting roots to grow downward and acquire water from deeper soil. In the present study, we found that *ZmDRO1* responded more strongly to abscisic acid (ABA)/drought induction in *Zea mays* ssp*. mexicana*, an ancestral species of cultivated maize, than in B73. It was proposed that this is one of the reasons why *Zea mays* ssp*. mexicana* has a more noticeable change in the downward direction angle of the root and fewer biomass penalties under water‐deficient conditions. Thus, a robust, synthetic ABA/drought‐inducible promoter was used to control the expression of *ZmDRO1* ^*B73*^ in *Arabidopsis* and cultivated maize for drought‐resistant breeding. Interestingly, ABA‐inducible *ZmDRO1* promoted a larger downward root angle and improved grain yield by more than 40% under water‐limited conditions. Collectively, these results revealed that different responses to ABA/drought induction of *ZmDRO1* confer different drought avoidance abilities, and we demonstrated the application of *ZmDRO1* via an ABA‐inducible strategy to alter the root architecture of modern maize to improve drought adaptation in the field.

## Introduction

Maize (*Zea mays* ssp. *mays* L.), one of the most important food crops in the world, is highly sensitive to water deficiency, especially during flowering, pollination and embryo development (Boyer and Westgate, 2004; Xu *et al*., 2014). When exposed to drought stress, plants avoid production loss using different strategies to cope with adverse factors, such as, drought avoidance, desiccation prevention and rapid recovery of growth following rewetting (Comas *et al*., 2013; Gowda *et al*., 2011). Among these mechanisms, roots are most likely to be associated with drought avoidance, as root systems are the most crucial components responsible for water acquisition. Dryland plants usually have longer and deeper roots than hygrophytes, and therefore have better access to water resources in deep soil (Comas *et al*., 2013; Gowda *et al*., 2011; Merrill *et al*., 2002; Moroke *et al*., 2005). Root architecture remodelling (RAR) significantly affects crop yields and biomass in dry environments. Enhanced hydrotropism of maize roots has been reported to correlate with better adaptation to drought and partial lateral irrigation, indicating that selection for robust hydrotropism may be a promising breeding strategy to improve drought avoidance in maize (Eapen *et al*., 2017). *DEEPER ROOTING 1* (*OsDRO1*), a quantitative trait locus (QTL) mapping identified gene from an upland rice cultivar, could increase the root growth angle and enable the recipient cultivar to avoid drought stress by increasing deep rooting, and subsequently maintain a high yield performance under drought conditions (Guseman *et al*., 2017; Uga *et al*., 2013).

The domestication of maize was proposed to have started with Balsas teosinte (*Zea mays* ssp. *parviglumis*) approximately 9000 years ago in southern Mexico (Heerwaarden *et al*., 2011; Piperno *et al*., 2009). A recent study showed that the modern maize genome contains over 10% introgression from the *Zea mays* ssp. *mexicana* (hereafter, *mexicana*) genome (Yang *et al*., 2017) and gene flow from *mexicana* has contributed to maize local adaptation and improvement (Heerwaarden *et al*., 2011; Yang *et al*., 2017), and thus *mexicana* is an important ancestor of modern maize. Although teosintes have several traits unsuitable for cultivation and consumption, multiple resistance traits are beneficial for maize adaptation (Burton *et al*., 2013; Chen *et al*., 2015a; de Lange *et al*., 2014). Long‐term breeding of crops for maize production has produced a suite of desirable traits suited to human needs and adapted to the conditions of cultivation. However, many teosinte resistance traits to specific environmental conditions, such as edaphic stress, pathogens, and pest pressures, have been lost in modern maize (Burton *et al*., 2013; Chen *et al*., 2015a; de Lange *et al*., 2014). Modern maize cultivars have different root architectures, particularly the root growth angle (RGA), compared to ancestral species (Burton *et al*., 2013; Omori and Mano, 2007). A study investigating the major QTL regulating RGA was performed in F_2_ populations of maize ‘B73’ × teosinte ‘*Zea luxurians*’, and several major QTL have been identified (Omori and Mano, 2007). Interestingly, one major QTL located at the terminal of the long arm of chromosome 7 harbours the highest homologous gene of *OsDRO1*, called *ZmDRO1*.

Abscisic acid (ABA) is important for the amelioration of drought stress. ABA accumulates rapidly and plays a positive role in drought tolerance by regulating multiple processes at different tiers, such as the expression of ABA‐responsive genes, stomatal closure, RAR, and the production of protective metabolites (Cao *et al*., 2013; Harris, 2015; Mehrotra *et al*., 2014; Zhang *et al*., 2006). Because of their general behaviour and broad effects, ABA signalling cascades give rise to many strategies for the engineering of crop tolerance to drought, for example manipulation of ABA receptors (Miaoa *et al*., 2018), exploration of ABA analogues (Cao *et al*., 2017), and utilization of ABA‐induced promoters to drive some genes that are beneficial for drought tolerance (Chen *et al*., 2015b).

In this study, *ZmDRO1* was proposed to contribute directly to the differences in root angle and drought avoidance between B73 and *mexicana*. This may be because *ZmDRO1* plays a positive role in drought avoidance and displays a lower basal expression, but a stronger response to ABA induction in *mexicana* than in B73. Based on the mechanism of drought avoidance, a synthetic ABA‐inducible promoter was used to drive the expression of *ZmDRO1* in modern maize, and successfully improved grain yield in the field under water‐limited conditions.

## Results

### *ZmDRO1* likely contributes to the differences in root growth angle and drought avoidance between B73 and *mexicana*

*OsDRO1* enhances drought avoidance by increasing the downward root growth angle of rice to draw deeper soil water and reduce the yield penalty under water‐limited conditions (Uga *et al*., 2013). The highest homologous gene of *OsDRO1* in maize is GRMZM2G700200 (Figure S1a), and it is located in a major QTL that may determine the different root growth angles between B73 and teosinte *Z. luxurians* (Omori and Mano, 2007). Hereafter, GRMZM2G700200 is referred to as *ZmDRO1*. B73 and *mexicana* also have very different root architectures, particularly the root growth angle. First, we compared the drought avoidance of B73 and *mexicana*. Seedlings were cultivated in plastic tubes (diameter × depth = 30 × 60 cm) and exposed to water‐limited conditions. Shoot dry weight, root dry weight and the distribution of root biomass in three different soil layers (<10, 10–20, and >20 cm) were measured to reflect drought avoidance. B73 plants had more biomass under well‐watered conditions but similar biomass under water‐deficient conditions compared to *mexicana* (Figure S1a,b), implying that *mexicana* had less biomass penalty and was more tolerant to drought stress. Under water‐deficient conditions, root architecture was remodelled. The proportion of deeper roots decreased in B73 and increased in *mexicana* (Figure S1c), indicating that *mexicana* plants could better adapt to water‐deficient conditions by increasing downward root growth.

**Figure 1:**
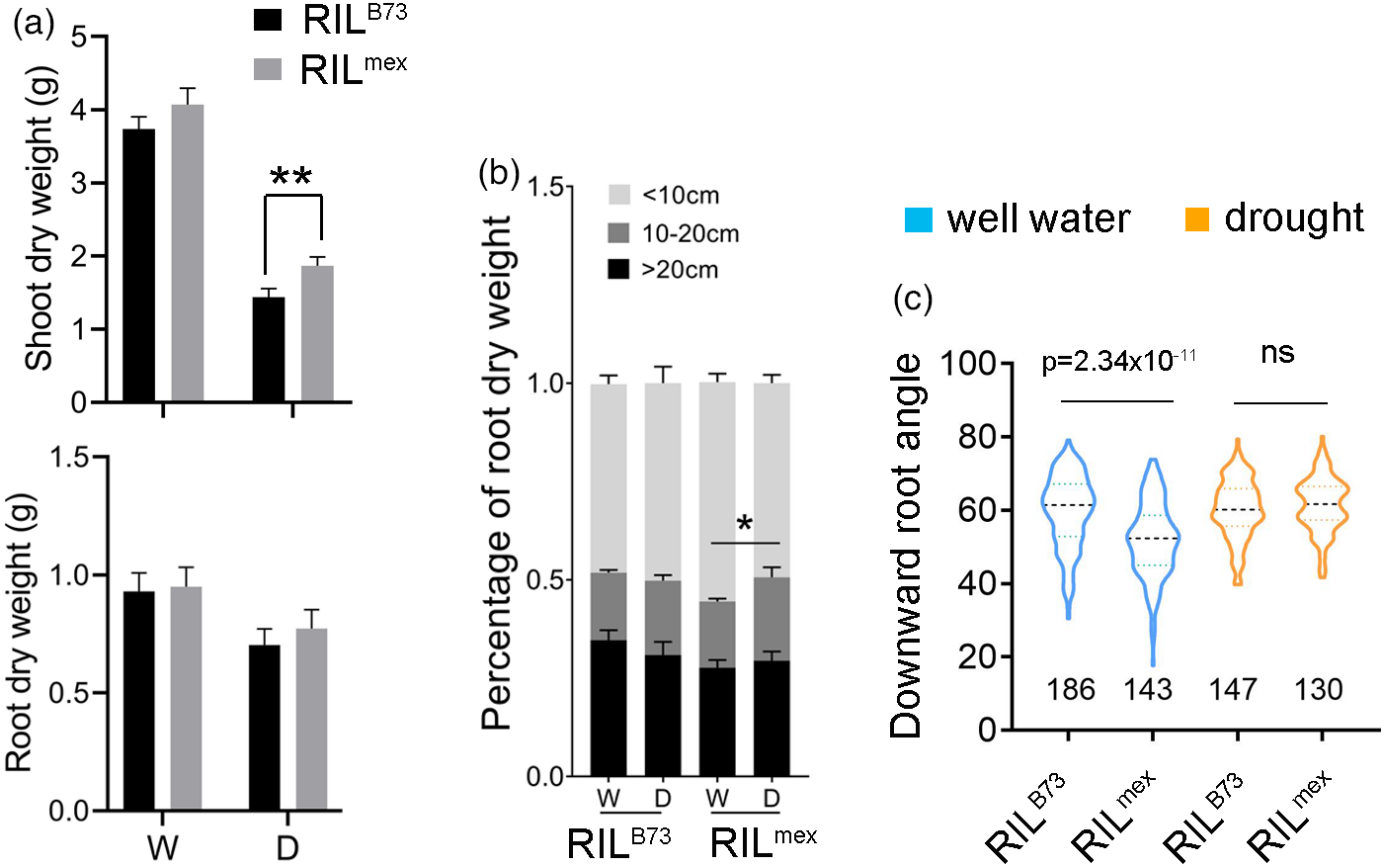
*ZmDRO1* may contribute to the different root growth angle and drought avoidance between B73 and *mexicana*. (a) Biomass and (b) root distribution of recombinant inbred lines (RILs) with *ZmDRO1* ^*B73*^ (RIL^B73^) or *ZmDRO1* ^*mex*^ (RIL^mex^) grown in plastic tubes (diameter × depth = 30 × 60 cm) under well‐watered (W) or drought‐stress (D) conditions. (c) Downward root growth angle of RILs under well‐watered or drought‐stress conditions in field. Statistical analysis was performed with Student's *t‐test*. Asterisks on bar represent the difference is significant (*P < 0.05, **P < 0.01). 30 and 45 plants, respectively, of RIL^mex^ and RIL^B73^ were investigate in (a) and (b). The number below the violin in figure c indicates the number of plants.

To verify the role of *ZmDRO1* in drought avoidance, the BC_2_F_8_ population of ‘B73 × *mexicana*’ was developed. Given the unknown sequence of *ZmDRO1* ^*mex*^, genome walking was used to obtain the DNA sequence of *ZmDRO1* ^*mex*^, extending from the conservative coding region. The whole gene, with a 345‐bp sequence upstream of the translation initiation site, was finally identified. The noncoding region upstream of the second intron was remarkably different between B73 and *mexicana*, whereas the amino acid sequence was conserved (Figures S1d and S2a). Based on the difference in DNA sequence between *mexicana* and B73, a molecular marker was designed for genotyping in the BC_2_F_8_ population (Figure S2b). Ten ZmDRO1^mex^ progenies (referred to as RIL^mex^) were identified from 108 progenies, and the remaining progenies were ZmDRO1^B73^ (referred to as RIL^B73^). Fifteen randomly selected RIL^B73^ and 10 RIL^mex^ progenies were cultivated in plastic tubes (diameter × depth = 30 × 60 cm) and exposed to water‐limited conditions. Root biomass was comparable between RIL^B73^ and RIL^mex^ under both well‐watered and water‐deficient conditions (Figure 1a). Shoot biomass was comparable between RIL^B73^ and RIL^mex^ under well‐watered conditions, but higher in RIL^mex^ under water‐deficient conditions (Figure 1a), implying that RIL^mex^ may be more tolerant to drought stress. Under water‐deficient conditions, the proportion of the deeper roots was unchanged in RIL^B73^ and increased in RIL^mex^ (Figure 1b). Furthermore, 25 progenies were cultivated in the field and initially exposed to mild water‐deficient conditions at the eight‐leaf stage. The downward root growth angle (shown in Figure S2c) was larger in RIL^B73^ than in RIL^mex^ under well‐watered conditions, and it increased under water‐deficient conditions in RIL^mex^ plants but not in RIL^B73^ plants (Figure 1c). These results confirmed that *ZmDRO1* contributed to the different root growth angles and drought avoidance between B73 and *mexicana*.

The genome sequences of *ZmDRO1* ^*mex*^ and *ZmDRO1* ^*B73*^ were used for BLAST, referring to the genomic information of various inbred lines in the MaizeGDB database. Thirty‐eight inbred lines were similar to B73, and just two inbred lines (CML333 and teosinte PI566673 Yan) were similar to *mexicana* (Figure S2d). Thus, the genotype of *ZmDRO1* ^*mex*^ may exist in teosinte and has been lost in most cultivated maize during domestication.

### *ZmDRO1* ^*B73*^ and *ZmDRO1* ^*mex*^ are differentially induced by ABA

**Figure 2:**
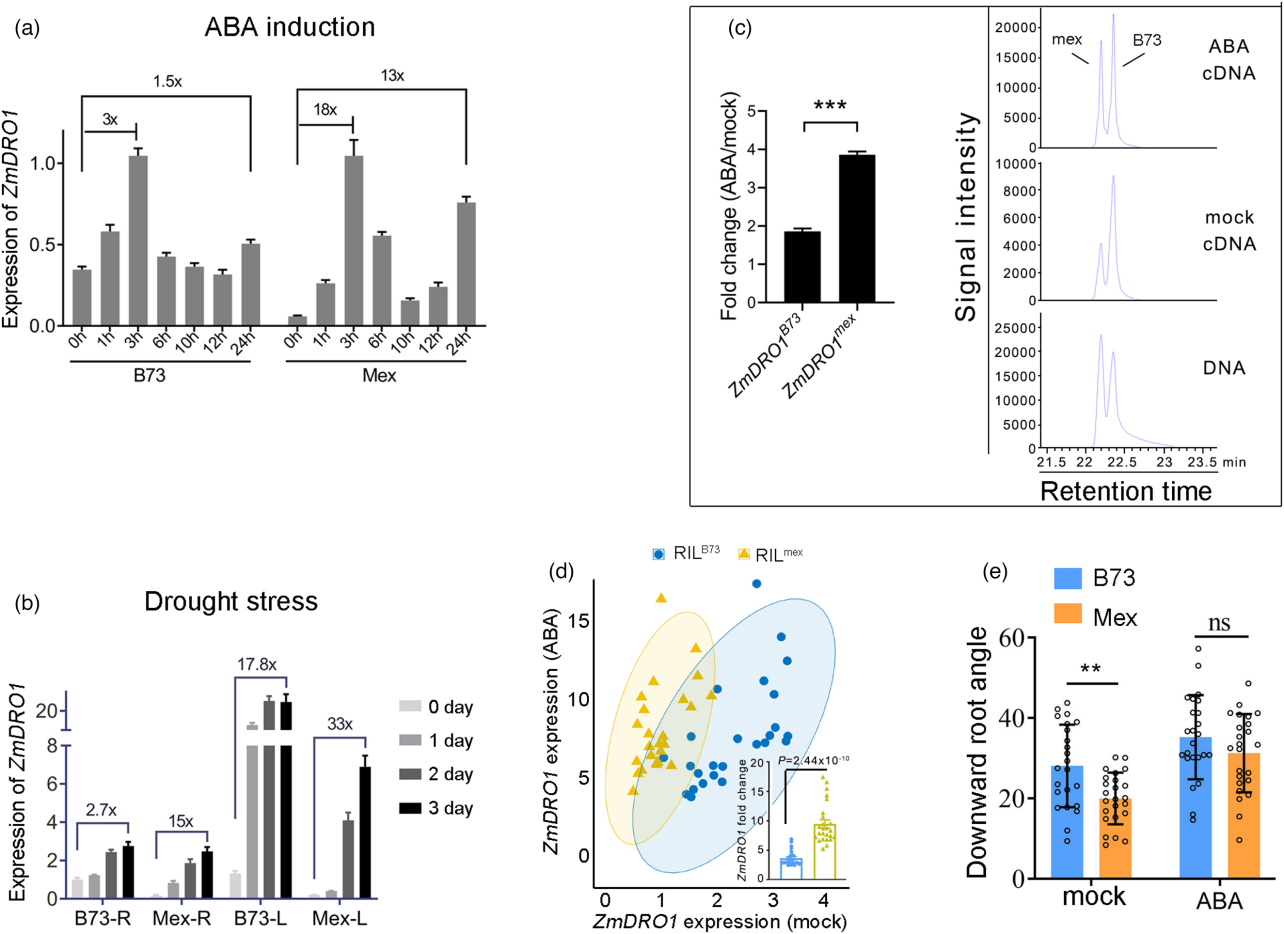
*ZmDRO1* ^*B73*^ and *ZmDRO1* ^*mex*^ are differentially induced by ABA. (a) Expression of *ZmDRO1* in roots of B73 and *mexicana* under ABA (50 μM) treatment. (b) Expression of *ZmDRO1* in roots (R) and leaves (L) of B73 and *mexicana* under drought stress. *ZmGAPDH1* and *ZmeF1α* were used as reference genes to normalize the expression of *ZmDRO1*. (c) Expression of *ZmDRO1* ^*B73*^ and *ZmDRO1* ^*mex*^ in roots of hybrid plants (RIL^B73/mex^) under ABA (50 μM) treatment for 3 h was tested by using genetic analysis system. The peak area of DNA was used to normalize the peak of cDNA and the changes in expression levels after ABA treatment was showed as bar chart. This experiment has been repeated three times. (d) Expression of *ZmDRO1* in roots of RILs with *ZmDRO1* ^*B73*^ or *ZmDRO1* ^*mex*^ under ABA (50 μM) treatment. One dot represented a plant. (e) The downward root growth angle of B73 and *mexicana* under mock and ABA (50 μM) treatment. Statistical analysis was performed with Student's *t*‐test. Asterisks on bar represent the difference is significant (*P < 0.05, **P < 0.01).

Given that the noncoding region upstream of the second intron of *ZmDRO1* is remarkably different between B73 and *mexicana*, and the amino acid sequence is conserved, the four amino acid substitutions of ZmDRO1 between B73 and *mexicana* were analysed. Multiple sequence alignment of orthologous genes of *DRO1* from 18 species showed that all four amino acid substitutions in ZmDRO1 between B73 and *mexicana* were not conserved (Figure S3a). In line with this, substitutions between B73 and *mexicana* were predicted to be neutral (http://www.ppved.org.cn/) (Figure S3b) (Gou *et al*., 2022). Therefore, the different effects of *ZmDRO1* ^*B73*^ and *ZmDRO1* ^*mex*^ may be resulting from the transcriptional level. In rice, *OsDRO1* was reported to work downstream of IAA signalling, was rapidly down‐regulated by IAA treatment, and was proposed to be regulated by the binding of auxin response factors (ARFs) to TGTCTC and TGTC motifs (Uga *et al*., 2013). Therefore, the transcriptional response of *ZmDRO1* to IAA, GA3, ABA, and ethephon was investigated in the seedling roots. *ZmDRO1* displayed the most dramatic response to ABA in both B73 and *mexicana. ZmDRO1* had a lower basal expression but a stronger response to ABA induction in *mexicana* than in B73 (Figure 2a). IAA, GA3, and ethephon weakly down‐regulated *ZmDRO1* in B73 but did not affect *mexicana* (Figure S4a). The promoters of *ZmDRO1* from B73 (896 bp) and *mexicana* (896 bp) were analysed. There were five TGTC motifs but no TGTCTC motifs in *DRO1* ^*B73*^ (Figure S4b). However, there were two TGTC and one TGTCTC motifs in *DRO1* ^*mex*^ (Figure S4b). In addition, two ABA responding motifs were identified in the promoters of *OsDRO1* and *ZmDRO1* ^*B73*^, and one was identified in *ZmDRO1* ^*mex*^ (Figure S4b). The difference in the promoters may explain the different response between *ZmDRO1* ^*B73*^ and *ZmDRO1* ^*mex*^ to ABA stimuli. However, the effect of the large difference in the second intron of *ZmDRO1* between B73 and *mexicana* cannot be ruled out. B73 and *mexicana* plants were further cultivated in soil and exposed to drought stress. Similarly, *ZmDRO1* of *mexicana* plants displayed a larger fold change than that of B73 in both roots and shoots under drought stress (Figure 2b). This was consistent with the transcriptome data from a previous report that *ZmDRO1* was induced by drought stress (Figure S4c; Liu *et al*., 2020). To further confirm the different responses to ABA stimuli between *ZmDRO1* ^*B73*^ and *ZmDRO1* ^*mex*^, RIL^B73^ and RIL^mex^ were used for transcriptional investigation by using qPCR, and heterozygous plants (*ZmDRO1* ^*B73/mex*^) were used for transcript level comparison using a genetic analysis system. Similarly, *ZmDRO1* ^*mex*^ displayed lower basal expression but a stronger response to ABA induction than *ZmDRO1* ^*B73*^, both in different and in the same genetic background (Figure 2c,d). These lines of evidence indicate that variations in *ZmDRO1* were the cause of different responses to ABA between B73 and *mexicana*.

Given the effect of *DRO1* on promoting downward root growth, roots of B73 and *mexicana* were transplanted horizontally in sands and treated with ABA. After 12 h of growth under mock or ABA treatment, the downward root growth angle (the angle between the root tip and horizontal ground) was measured. Under mock conditions, B73 had a larger root angle than that of *mexicana* (Figure 2e). The root angle increased in both B73 and *mexicana* after ABA treatment, and it increased more in *mexicana* (Figure 2e). This is consistent with the root angle phenotype of RIL^B73^ and RIL^mex^ in the field (Figure 1c).

In addition, the expression pattern of *ZmDRO1* in different tissues was investigated in B73 and *mexicana. ZmDRO1* displayed a similar expression pattern in B73 and *mexicana*, with the highest level in the coleoptile (Figure S4d). *ZmDRO1* was higher expressed in B73 than in *mexicana* in most organs, but was lower expressed in the ear and husk (Figure S4d). *ZmDRO1* ^*B73*^ *‐GFP* and *ZmDRO1* ^*mex*^ *‐GFP* were transiently expressed in tobacco leaf and maize protoplasts to determine their subcellular localization. ZmDRO1^B73^‐GFP and ZmDRO1^mex^‐GFP were mainly located on the membrane and some were distributed in the cytoplasm (Figure S4e).

### Effect of ABA‐inducible *ZmDRO1* on *Arabidopsis* root growth

Given that *ZmDRO1* ^*mex*^ may improve the drought avoidance of plants more effectively than *ZmDRO1* ^*B73*^, with a stronger response to ABA induction, we speculated that a more powerful ABA‐inducible promoter may increase the contribution of *ZmDRO1* to drought avoidance, and this strategy would reduce the possible adverse effects of constitutive overexpression under optimal conditions. It is difficult to obtain a longer promoter for *ZmDRO1* ^*mex*^. Therefore, a synthetic ABA‐inducible promoter, which maintains a low basal level of downstream gene expression under normal growth conditions, but confers a high level of expression under ABA induction (Chen *et al*., 2015b), was modified and used to drive *ZmDRO1* ^*B73*^ in *Arabidopsis* and maize (Figure S5a,b). As expected, the basal expression level of most transgenic lines was low and slightly higher than that of the maize recipients (Figure S5d). Upon ABA treatment, most transgenic lines had a significantly higher expression level of *ZmDRO1* than the non‐transgenic plants (Figure S5c–e). However, some abnormal lines were also observed, in which *ZmDRO1* displayed high basal expression level or was not induced by ABA (Figure S5c–e). In addition, we found that the observed molecular weight of ZmDRO1 was larger than its theoretical molecular weight. Thus, ZmDRO1 may have some protein modifications.

**Figure 3:**
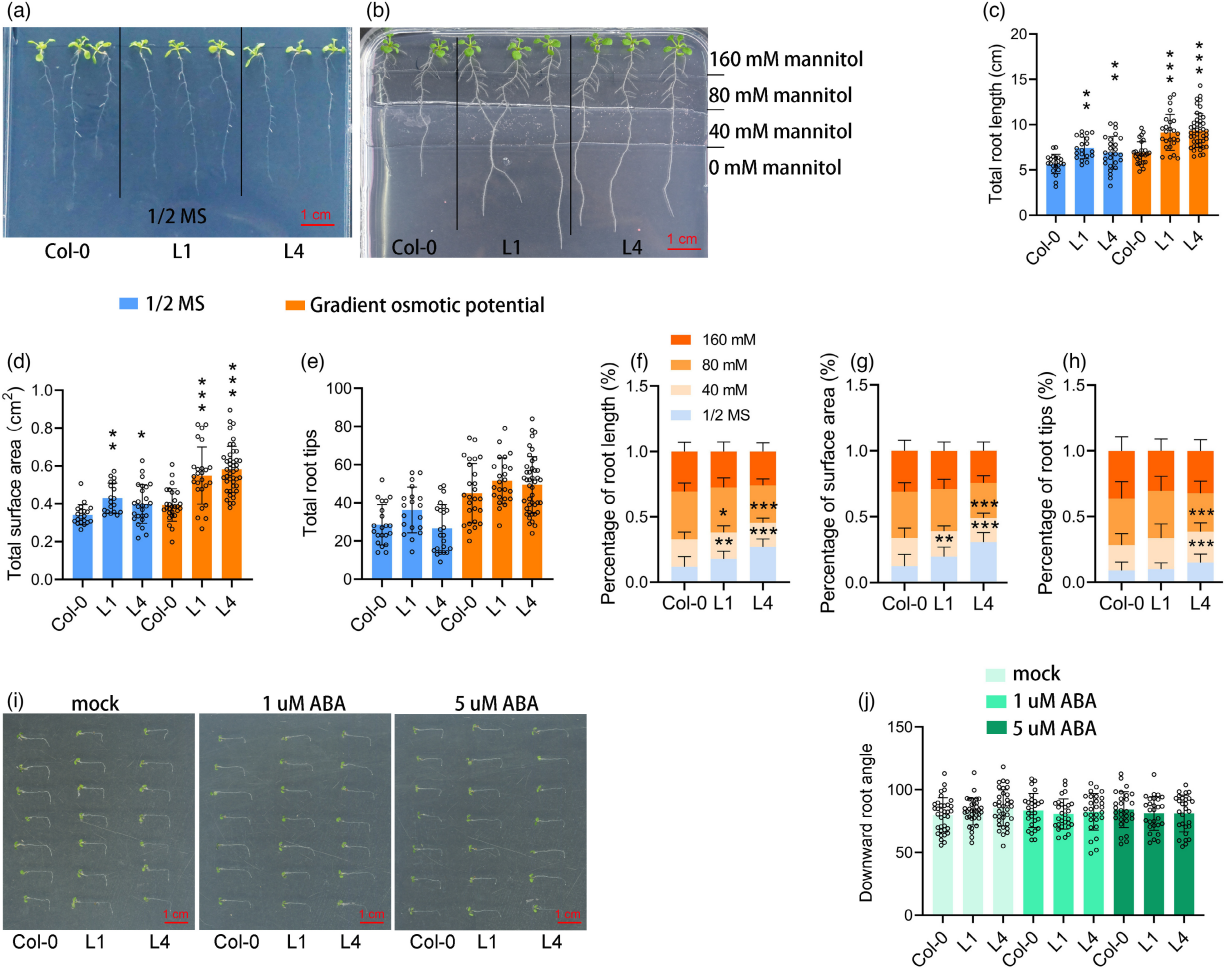
Effect of ABA‐inducible *ZmDRO1* on *Arabidopsis* root growth. (a, b) Arabidopsis were grown on 1/2 MS medium (16‐days old) or medium with hydraulic potential gradient produced by different concentrations of mannitol (20‐days old). (c–e) Three traits of root on 1/2 MS and hydraulic potential gradient medium. The percentage of root length (f), root surface area (g) and root tips (h) on hydraulic potential gradient medium in different concentrations of mannitol. Phenotype of root bending (i) and the statistic of bending angle (j) under ABA treatments. One circle represented a plant. Statistical analysis was performed with one‐way ANOVA, and the mean value of L1 and L4 was compared with Col‐0. Asterisks on bar represent the difference is significant (*P < 0.05, **P < 0.01, ***P < 0.001).

Transgenic *Arabidopsis* lines L1 and L4 were used for further study, and Col‐0 was used as the control line. Different concentrations of mannitol were used to prepare culture media to mimic the hydraulic potential gradient in the field, as shown in Figure 3b. Interestingly, transgenic plants grew remarkably better than Col‐0 plants, with longer root length and larger root surface area, both in 1/2 MS and gradient‐mannitol media (Figure 3a–e). Moreover, a greater proportion of the roots of transgenic plants could grow deeper to reach the 1/2 MS layer (Figure 3b,f–h). Furthermore, the downward root growth angle was measured after treatment with different concentrations of ABA. The bending of *Arabidopsis* root was rapid and sharp, with no difference between plants or treatments (Figure 3i,j).

### ABA‐inducible *ZmDRO1* improved drought avoidance in maize

**Figure 4:**
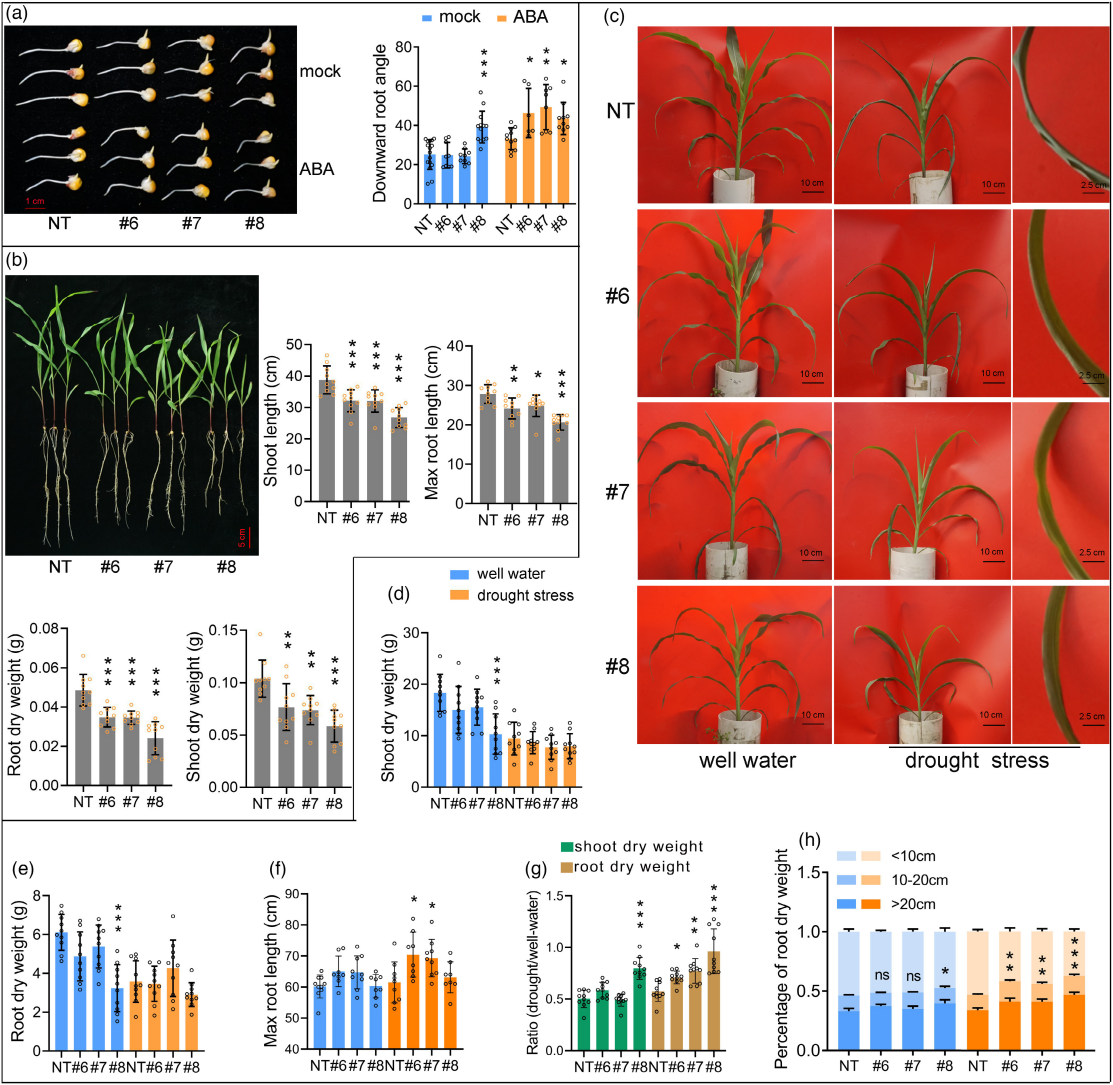
ABA‐inducible *ZmDRO1* improved drought avoidance of maize seedlings in artificial greenhouse. (a) Phenotype of root bending and the statistic of bending angle under ABA treatments (50 μM). (b) Phenotype and root traits of maize seedlings grown in rolled‐up germinating test paper in nutrient solution. (c–h) Biomass and root traits of maize seedlings grown in plastic tubes (diameter × depth = 30 × 60 cm) under well‐watered or drought‐stress conditions. One circle represented a plant. Statistical analysis was performed with one‐way ANOVA, and the mean value of #6, #7 and #8 was compared with NT. Asterisks on bar represent the difference is significant (*P < 0.05, **P < 0.01, ***P < 0.001).

Two low basal expression lines (#6 and #7), one constitutive high expression line (#8), and a segregated non‐transgenic plant (NT) were used for further study (Figure S5d,e). First, the downward root bending was tested. After 12 h of growth under mock or ABA treatment, the downward root growth angle was measured. As expected, ABA stimuli promoted downward root growth, and the promotion effect was enhanced by ABA‐inducible *ZmDRO1* (Figure 3a). Additionally, line #8 had a large downward root angle under the mock treatment and was not promoted by ABA treatment (Figure 3a). Seedlings were grown in rolled‐up germinating test paper in a nutrient solution, and the growth of transgenic plants, especially line #8, was weaker than that of NT plants (Figures 4b and S6). Seedlings were then grown in plastic tubes (diameter × depth = 30 × 60 cm). Similarly, the growth of line #8 was weaker than that of NT plants under well‐watered conditions (Figure 4c–e). The growth vigour of lines #6 and #7 was comparable to that of NT plants (Figure 4c–e). Interestingly, the degree of leaf wilting and the reduction in biomass was significantly less in transgenic plants than in NT plants under drought stress (Figure 4c,g). Moreover, the ABA‐inducible transgenic plants (#6 and #7) grown in soil did not show a reduction in root length and had longer roots than NT plants under drought stress (Figure 4f). The distribution of root biomass in three different soil layers (<10, 10–20, and >20 cm) showed that ABA‐induced *ZmDRO1* could improve the percentage of deeper roots under water‐deficient conditions (Figure 4g).

**Figure 5:**
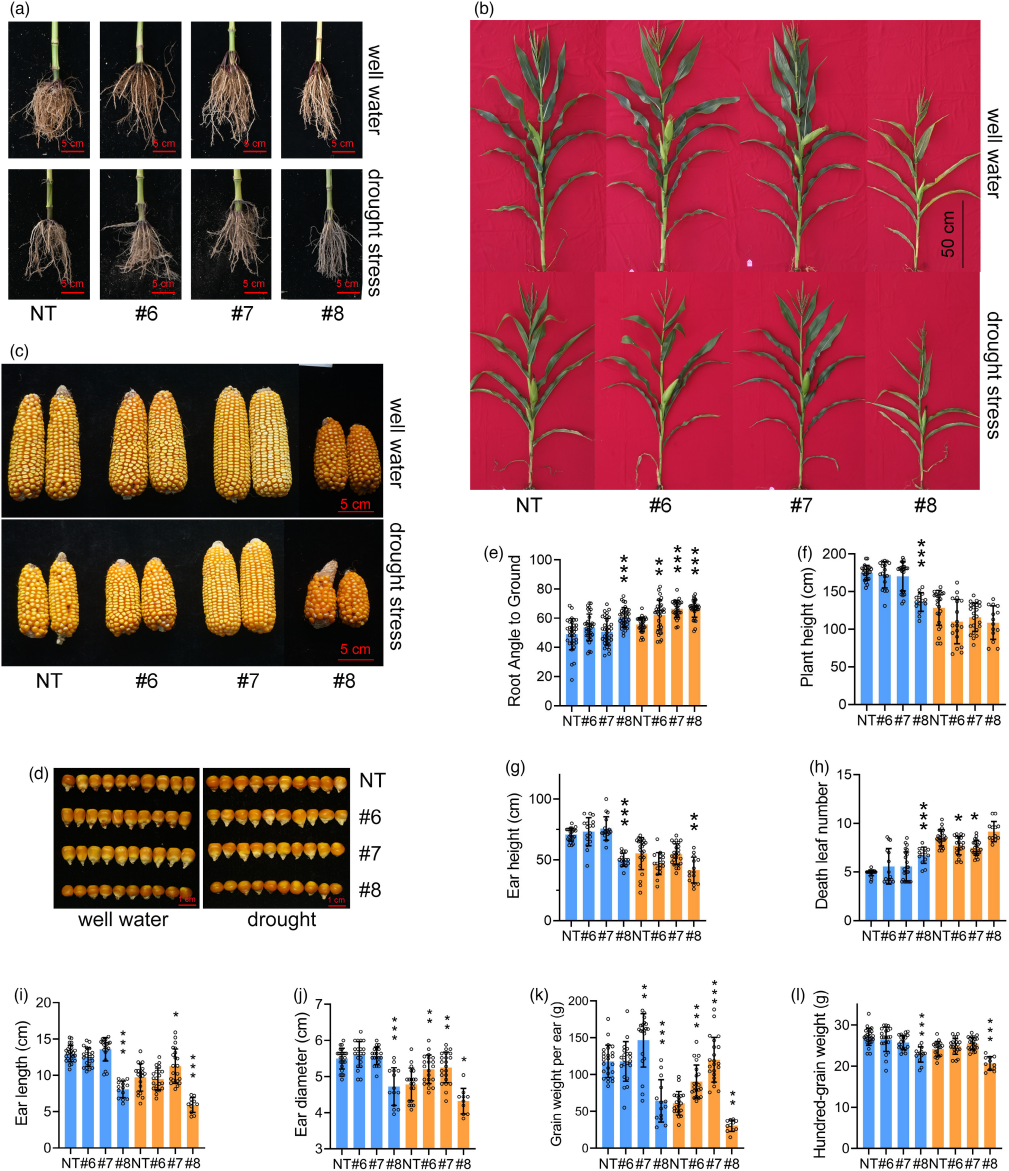
ABA‐inducible *ZmDRO1* improved drought adaption of maize in field. Phenotype of root (a), shoot (b), ear (c) and seed (d) under well‐watered or drought‐stress conditions. (e–l) The statistic of agronomic traits and yield traits under well‐watered or drought‐stress conditions. One circle represented a plant. Statistical analysis was performed with one‐way ANOVA, and the mean value of #6, #7 and #8 was compared with NT. Asterisks on bar represent the difference is significant (*P < 0.05, **P < 0.01, ***P < 0.001).

Based on the above results and the phenotypes of #8 and #10 in the field, we propose that constitutively high expression of *ZmDRO1* severely represses plant growth (Figures 5b,c,f‐i and S7a,b,d,g). ABA‐inducible *ZmDRO1* did not affect most of the visible agronomic traits under well‐watered conditions, except for the promotion of flowering (Figures 5e–h and S7c–h). The ABA‐inducible transgenic plants (#6 and #7) had larger downward root growth angles, few dead leaves, and higher grain yield under drought stress conditions (Figure 5d). The grain yield of ABA‐inducible transgenic plants was higher than that of NT plants under drought stress at four different locations, and the increase in grain yield was more than 40% (drought stress was too severe in Xinjiang in 2020) (Table 1).

**Table 1:** Grain yield per year in fields in different years (g)

Year‐field	Samples	Well watered (mean ± SD)	Drought stress (mean ± SD)
2019‐Hainan	NT	118 ± 21.9	61 ± 16.0
	#6	118 ± 26.9	91 ± 22.4***
	#7	147 ± 36.2**	120 ± 30.3***
	#8	64 ± 28.8***	31 ± 7.7**
2020‐Xinjiang	NT	76 ± 13.8	18 ± 10.8
	#6	74 ± 15.3	26 ± 20.3
	#7	85 ± 18.2	30 ± 13.6*
2020‐Hainan	NT	109.2 ± 15.7	34 ± 16.1
	#6	114 ± 28.8	62 ± 29.9*
	#7	135 ± 35.9*	66 ± 23.4***
2021‐Hainan	NT	98 ± 12.6	57 ± 16.9
	#6	106 ± 28.4	83 ± 37.5**
	#7	130 ± 52.0*	121 ± 39.1***

Statistical analysis was performed with one‐way ANOVA, and the mean value of #6, #7 and #8 was compared with NT. Asterisks on bar represent the difference is significant (*P < 0.05, **P < 0.01, ***P < 0.001).

### ABA‐inducible transgenic maize has low water use efficiency

**Figure 6:**
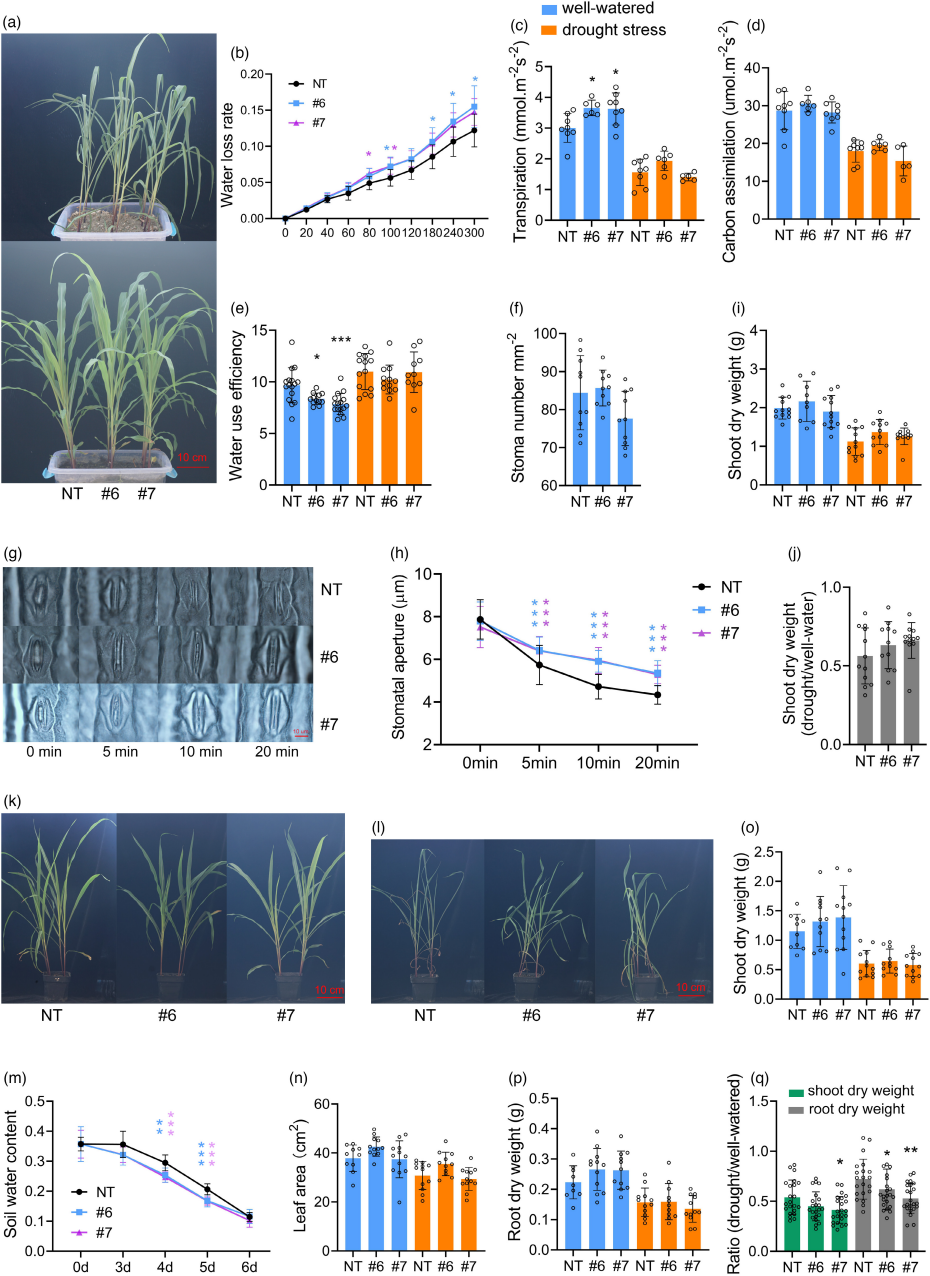
*ZmDRO1* will decrease water use efficiency with higher transpiration rate. (a–j) The phenotype of maize seedlings grown together in basins (length × width × depth = 35 × 25 × 15 cm) with 13‐cm soil layer under well‐watered and drought stress conditions. (b) The water loss rate of detached leaf. Eighteen pieces of detached leaf of each sample were tested here. Transpiration rate (c) and carbon assimilation rate (d) were measured by photosynthesis measurement system, and water use efficiency (e) was calculated from transpiration rate and carbon assimilation rate. (f) Stoma intensity on the fourth abaxial leaf under well‐watered conditions. (g–h) The change of stomatal aperture after detachment was measured from 10 leaves of each sample. Shoot dry weight (i) and ratio (shoot dry weight: drought/well‐watered) (j) of maize seedlings. (k–q) The phenotype of maize seedlings grown individually in pots of 13‐cm depth with the same amount of soil (length × width × depth = 10 × 10 × 13 cm) under well‐watered (k) and drought stress (l) conditions. (m) The change of soil water content after the halting of watering about 18‐day‐old plants. Five pots of each sample were measured. The mean of different sample at 0‐point was normalized to the same. (n) The third leaf area of 23‐day‐old plants. (o) Shoot dry weight. (p) Root dry weight. (q) Ratio of dry weight (drought/well‐watered).

If the soil layer is thin, what is the difference in drought tolerance between ABA‐induced transgenic plants and NT plants? To better understand the role of ABA‐inducible *ZmDRO1*, lines #6, #7 and NT were grown together in basins (length × width × depth = 35 × 25 × 15 cm) with a 13‐cm soil layer. Similar to the seedlings grown in plastic tubes, the degree of leaf wilting in #6 and #7 was lower than that in the NT plants under drought stress conditions (Figure 6a). Thus, the water loss rate of the detached leaves was measured. Interestingly, #6 and #7 displayed remarkably higher water loss rates than NT plants (Figure 6b). Moreover, #6 and #7 had higher transpiration rates, similar carbon assimilation rates and lower water use efficiencies than NT under well‐watered conditions (Figure 6c–e). This was due to the retardation of stomatal closure during dehydration rather than an increase in stomatal density (Figure 6f–h). In this condition, biomass penalties caused by drought stress for #6 and #7 were comparable to that for NT plants (Figure 6i,j).

In addition, NT, #6 and #7 were grown individually in pots of 13‐cm depth with the same amount of soil (length × width × depth = 10 × 10 × 13 cm). As expected, the decrease of soil water content in #6 and #7 was more rapid than that in NT plants, which was caused by the higher transpiration rate, but not the change in leaf area (Figure 6m,n). In this condition, drought stress caused a larger biomass penalty for #6 and #7 than for NT plants.

## Discussion

The role of *DRO1* in drought avoidance has been reported in several previous studies (Guseman *et al*., 2017; Uga *et al*., 2013). *OsDRO1* is negatively regulated by auxin signals to promote the downward growth of roots (Uga *et al*., 2013). In this study, we found that *ZmDRO1* ^*mex*^ had a stronger response to the induction of ABA and drought than *ZmDRO1* ^*B73*^, and it conferred a more remarkable change in the downward angle of the root and a lower penalty of biomass under water‐deficient conditions. With the application of a synthetic ABA‐inducible promoter to drive *ZmDRO1* in cultivated maize, the grain yield increased significantly under water‐deficient conditions and did not decrease under well‐watered conditions.

In maize, trait‐associated single nucleotide polymorphisms were reported to be enriched in nongenic regions, particularly within a 5‐kb window upstream of genes (Li *et al*., 2012). Recently, variations in promoters have been widely reported to alter gene expression and confer phenotypic variations (Li *et al*., 2017; Mao *et al*., 2015, 2022a,b; Tian *et al*., 2019; Wang *et al*., 2016). We found that the variations in the noncoding region upstream of the third exon between *ZmDRO1* ^*B73*^ and *ZmDRO1* ^*mex*^ were substantial. These variations are associated with the response of *ZmDRO1* to ABA/drought stimuli, downward root growth angle, and biomass penalty under drought stress conditions. Recombinant inbred lines harbouring *ZmDRO1* ^*mex*^ have a stronger response to ABA induction, larger downward root angle upon ABA stimulus, and higher grain yield in water‐deficient fields. Teosinte is the ancestral species of modern maize. Long‐term domestication of maize has produced many desirable traits suited to human needs and cultivation conditions. However, many teosinte resistance traits to specific environmental conditions have been lost in modern maize (Burton *et al*., 2013; Chen *et al*., 2015a; de Lange *et al*., 2014). Recently, a teosinte‐derived allele of *ZmMM1* was reported to positively regulate multiple disease resistance by repressing negative regulators of plant immunity (Wang *et al*., 2021). Based on the genome assembly of 40 inbred lines in public database MaizeGDB, we found that the genotype of *ZmDRO1* in 38 lines was similar to that of B73, and only two inbred lines were similar to *mexicana* (Figure S2d). Therefore, *ZmDRO1* ^*mex*^ may have been lost in most cultivated maize during domestication.

Inducible gene expression systems are favoured over stable expression systems in a wide variety of basic and applied research areas because constitutive gene expression generally has adverse effects (Chen *et al*., 2015b; Selvaraj *et al*., 2020). This was consistent with our finding that constitutively high expression of *ZmDRO1* severely represses maize growth. ABA/stress‐inducible promoters have been successfully used to drive the expression of positive regulators in drought tolerance, such as the late embryogenesis abundant protein gene Os*HVA1* and the CCCH‐tandem zinc finger protein gene *OsTZF5* (Chen *et al*., 2015b; Selvaraj *et al*., 2020). Given the stronger response of *ZmDRO1* ^*mex*^ to ABA/drought induction and its positive effect on drought avoidance, a synthetic ABA‐inducible promoter was used to drive *ZmDRO1B* ^*73*^ in cultivated maize as we did not obtain the complete promoter information of *ZmDRO1* ^*mex*^. ABA‐inducible *ZmDRO1B* ^*73*^ successfully increased deeper roots and grain yield under drought stress but did not cause yield loss under well‐watered conditions. However, the positive effect of ABA‐inducible *ZmDRO1B* ^*73*^ in drought adaptation was lost when roots were restricted to the shallow soil layer, and ABA‐inducible transgenic plants displayed lower water use efficiency than NT plants. Moreover, the higher transpiration rate of ABA‐inducible transgenic plants even increased their sensitivity to drought stress when seedlings were individually grown in 13‐cm deep pots. Therefore, the improved drought adaptation of ABA‐inducible transgenic plants in the field and plastic tubes was mainly due to an increase in deeper roots under drought stress. In addition to increasing transpiration rate, ABA‐inducible *ZmDRO1B* ^*73*^ also promote flowering. This may be caused by non‐tissue‐specific expression, as there was no expression of *ZmDRO1* in the ear leaf and stalk in the wild type. We speculate that a root‐specific ABA‐inducible promoter may have better performance.

Collectively, these results revealed that the different responses to ABA/drought induction of *ZmDRO1* confer plants with different ability in drought avoidance, and demonstrate the application of *ZmDRO1* via an ABA‐inducible strategy to alter the root architecture of modern maize to improve drought adaptation in the field.

## Experimental procedures

### Gene cloning and transformation

To clone *ZmDRO1* from *mexicana*, the genome walking method was used, and specific primers were designed in a conservative region in the third exon (Table S1). Primers SP1/2/3 were used with random primers for upstream sequence amplification, and primers SP4/5/6 were used with random primers for downstream sequence amplification. There was a large intron before the third exon. Thus, primers 7/8/9 were designed based on the amplified upstream sequence and were further used with random primers for upstream sequence amplification. Finally, the full‐length genome sequence and cDNA were amplified and verified by using sequencing.

The CDS of *ZmDRO1* ^*B73*^ was cloned using primers DRO1‐TF1/TR1 and fused with a Flag‐tag in an ABA‐inducible vector for transgenic plant creation (Feng *et al*., 2022). Primers DRO1‐GFP‐F/R1/R2 were used for the amplification of *ZmDRO1* ^*B73*^ and *ZmDRO1* ^*mex*^ to analyse subcellular localization using the vector pCAMBIA163‐1300. All primers used are listed in Table S1. Gene transformation for maize was performed at Weimi Biotechnology Co. LTD (Jiangsu, China) using the inbred line KN5585 as the recipient. *Arabidopsis* plants were transformed using the floral dip method. Transgenic plants were screened using 0.1% Basta (v/v), and verified using PCR. T3 homozygous transgenic *Arabidopsis* and wild‐type Col‐0 were used for phenotypic analysis. Transgenic maize was backcrossed with KN5585 twice before selfing for the homozygote. To minimize the influence of T‐DNA insertion on genes near the site, homozygous transgenic plants were backcrossed with KN5585, and the F1 hybrid progenies were used for phenotype analysis. Similarly, segregated non‐transgenic plants were backcrossed with KN5585, and F1 hybrid progenies were used as a non‐transgenic control (NT).

### Plants growth in different conditions and treatments

To analyse the distribution of roots in different soil depths, seedlings were grown in plastic tubes cut in half lengthwise with a 20‐cm diameter and 60‐cm depth. We stopped supplying water to the drought‐treated group after transplanting, and supplied sufficient water for the well‐watered group. When B73 or NT plants displayed a severe wilting state, the plastic tubes were opened, and roots in different depths of soil (<10, 10–20, and >20 cm) were collected.

For the drought stress test in shallow soil in separate pots, equal volumes of well‐mixed soil containing 200 mL of water were placed in each pot (length × width × depth = 10 × 10 × 13 cm), and four seedlings of each line were grown in different pots. Then, the tested group was watered quantitatively at approximately 12 days old and exposed to water outage until they displayed severe wilting, and the shoot dry weight, root dry weight, and leaf area were measured.

Basins were used for the drought stress test in shallow soil under the same conditions (length × width × depth = 35 × 25 × 15 cm). Various transgenic lines and NT plants were grown together in the same basin. When drought‐exposed seedlings displayed severe wilting, photosynthesis‐related traits, stomatal density, and shoot dry weight were measured. Maize was grown in a phytotron at 28 °C during the day and 25 °C at night. The illumination duration was 14 h, with an intensity of approximately 15 000 lux, and a relative humidity of about 60%. *Arabidopsis* was grown in a phytotron at 23 °C, with 14 h illumination and 3600 lux intensity.

For the field trials, plants were cultivated at Hainan in 2019, 2020, and 2021 (three replicates for each year), at Xinjiang in 2020 (four replicates). The average planting density was about 45 000/ha. Mild drought stress was managed by controlled irrigation at the 10‐leaf stage, and the NT plants displayed mild wilting.

To test the effect of ABA on root bending, maize seeds were germinated with approximately 1‐cm straight primary roots and then transplanted in sands horizontally, and 50 μm ABA was sprayed on the roots. After 12 h of growth under mock or ABA treatment, the downward root growth angle (the angle between the root tip and ground) was measured. For *Arabidopsis*, seeds germinated and grew vertically with a 1‐cm root on solid 1/2 MS medium. The seedlings were transferred to media containing different concentrations of ABA. The seedling roots were placed horizontally and grown for 24 h. The degree of root bending was then measured.

To analyse root traits, maize seedlings were grown in rolled‐up germination test paper in a nutrient solution. After 20 days, corresponding traits were analysed using WinRhizo Pro 2008a (Regent Instr. Inc., Quebec, Canada) with a professional scanner (Epson XL 1000, Japan). Maximum root length, root dry weight, shoot dry weight, and plant height (the length of the aerial part below the top phyllula) were measured.

### Gene expression analysis

Total mRNA was extracted and an equal amount of RNA of each sample was used for reverse transcription. Then an equal amount of cDNA of each sample was used for transcriptional level testing. Real‐time quantitative PCR was performed using SYBR Green Fast qPCR Mix (RM21203; ABclonal, Wuhan, China) and a Bio‐Rad CFX96 machine, California, USA. *ZmGAPDH1* and *ZmeF1α* were used as reference genes to normalize the expression of *ZmDRO1*. The difference between *ZmDRO1* ^*B73*^ and *ZmDRO1* ^*mex*^ in basal expression level and response to ABA stimuli in the same genetic background had to be more reliable. Therefore, heterozygous plants (*ZmDRO1* ^*B73/mex*^) were used for testing. The DNA and cDNA of *ZmDRO1* ^*B73/mex*^ was used as the template, and Cy5‐labelled primers were designed at two flanks of a polymorphic site (3‐bp indel). The Cy5‐labelled amplified fragments were used for capillary electrophoresis and the fluorescence signal of Cy5 was detected using a genetic analysis system (BECKMAN, GenomeLab GeXP, U.S.A.). The peak area of DNA was used to normalize the peak of cDNA, and the amplification cycle number was 30. The primers used are listed in Table S1. The protein level of ZmDRO1‐Flag in transgenic plants was tested using an automated Western blot system (Wes; ProteinSimple, California, USA), and the anti‐body for Flag‐tag was purchased from Merck (F3165, Darmstadt, Germany). Confocal laser microscopy (LSM800; Zeiss, Oberkochen, Germany) was used to investigate the subcellular localization of ZmDRO1‐GFP.

### Physiological traits and soil water content

At the five‐leaf stage, the middle parts of the fourth leaves were used for the stoma measurement. The number of stomata on the abaxial leaf was determined using an Olympus microscope (IX73, Tokyo, Japan) with a 10× objective lens. For each sample, 10 plants were selected for statistical analysis, and the experiment was repeated three times. To assess stomatal aperture during dehydration, the detached leaves were floated in stomatal opening buffer (10 mM Tris–HCl, pH 5.6, 10 mM KCl, and 50 μM CaCl_2_) for 3 h under light to induce the stomata to open to the maximum extent. Then, the surface water was wiped and detached leaves were placed on filter paper to induce stomatal closure, and the stomatal aperture was fixed at different time points using nail polish. The nail polish films were observed using an Olympus microscope (IX73), and images were collected using a 100× objective lens. Images were analysed using ImageJ software (National Institutes of Health, Maryland) to measure the aperture size. More than 30 stomata were measured per sample, and each sample included three replicates. To measure the water loss rate, detached leaves were placed on filter paper, and leaf weight was measured at different time points. Six detached leaves per sample were measured and each sample included three replicates. Photosynthesis‐related traits were measured using a photosynthesis measurement system (GFS‐3000; WALZ, Effeltrich, Germany). To analyse the soil moisture, 1‐cm^3^ of soil was collected from each pot at five different stages, beginning at about 18 days after planting (0, 3, 4, 5 and 6 days from the beginning point) and dried to calculate its water content. Five pots of each sample were measured. The mean of different samples at the 0‐day point was normalized.

### Accession numbers

*ZmDRO1*: GRMZM2G700200; *ZmGAPDH1*: GRMZM2G046804; *ZmeF1α*: GRMZM2G153541.

## Conflicts of interest

The authors declare that they have no competing interests.

## Authors’ contributions

XF designed the study. LJ, YC, XF, HG, DZ, WZ, HX, HZ, YW and XZ performed the main part of experiment. XF, YL and LJ analysed the data and wrote the article. YL, QW, JX and FW are responsible for managing materials. All authors read and approved of its content.

## Supporting information


**Figure S1** Teosinte *Zea mexicana* displayed better drought avoidance than B73.

**Figure S2** Gene sequence of *ZmDRO1* ^*mex*^ and allelic profile of *ZmDRO1* in RILs and inbred lines with reference genomic information.

**Figure S3** Amino acid sequences of ZmDRO1^B73^ and ZmDRO1^mex^ are highly similar.

**Figure S4** Expression pattern and subcellular localization of ZmDRO1^B73^ and ZmDRO1^mex^.

**Figure S5** Construction of ABA‐inducible expression cassette and the expression of various transgenic plants.

**Figure S6** Shoot and root phenotype of maize seedlings grown in rolled‐up germinating test paper in nutrient solution.

**Figure S7** Constitutive high expression of *ZmDRO1* will severely repress plant growth.

**Table S1** Primers used in this study.
