# Supplementary figures and images for: ABA‐inducible DEEPER ROOTING 1 improves adaptation of maize to water deficiency
Source: Plant Biotechnol J. 2022 Jul 22;20(11):2077–88. doi: 10.1111/pbi.13889 (PMC9616520; doi:10.1111/pbi.13889)

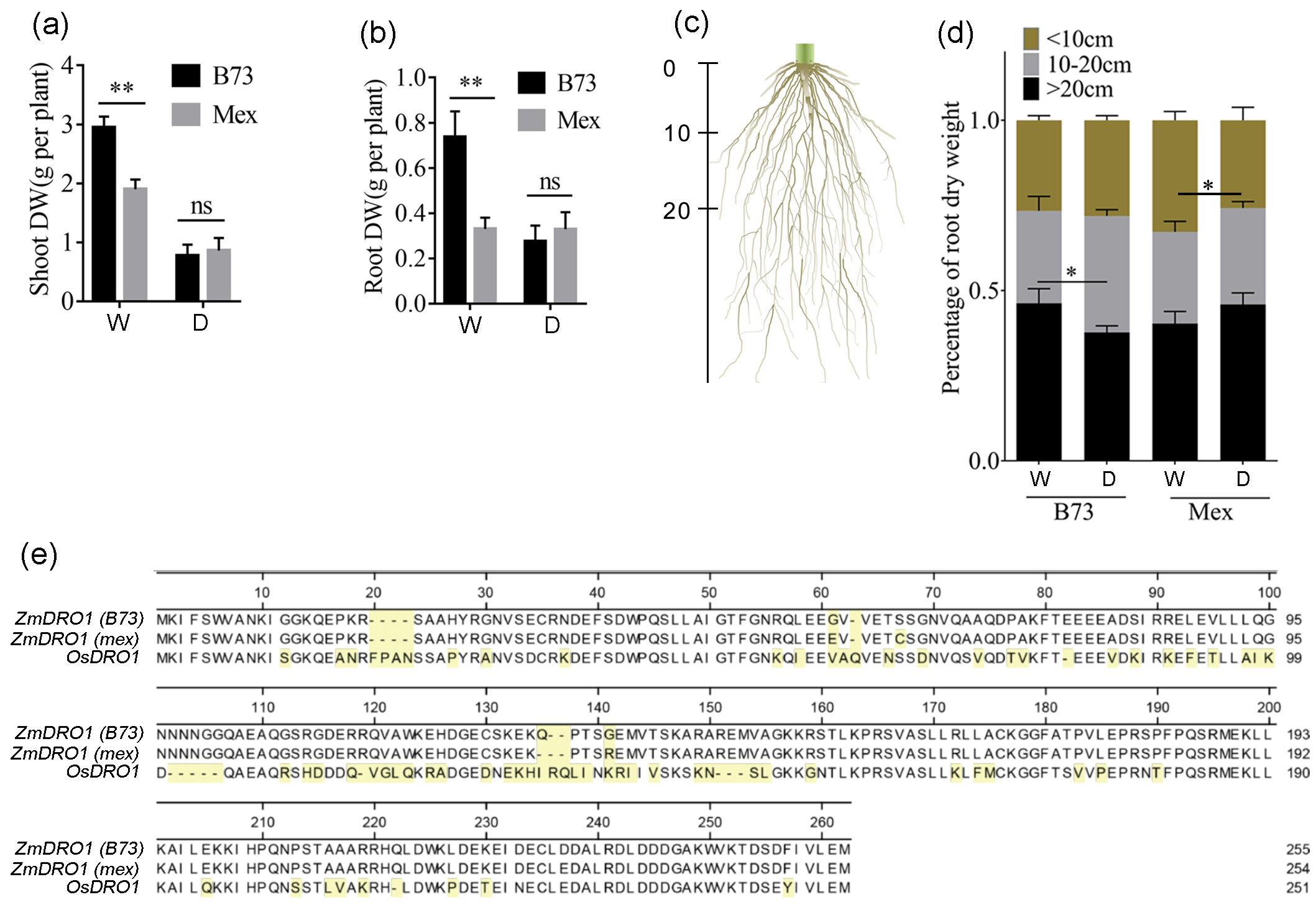

Supplement: Supplementary file 1 — Figure S1 Teosinte Zea mexicana displayed better drought avoidance than B73. [file PBI-20-2077-s006.tif]

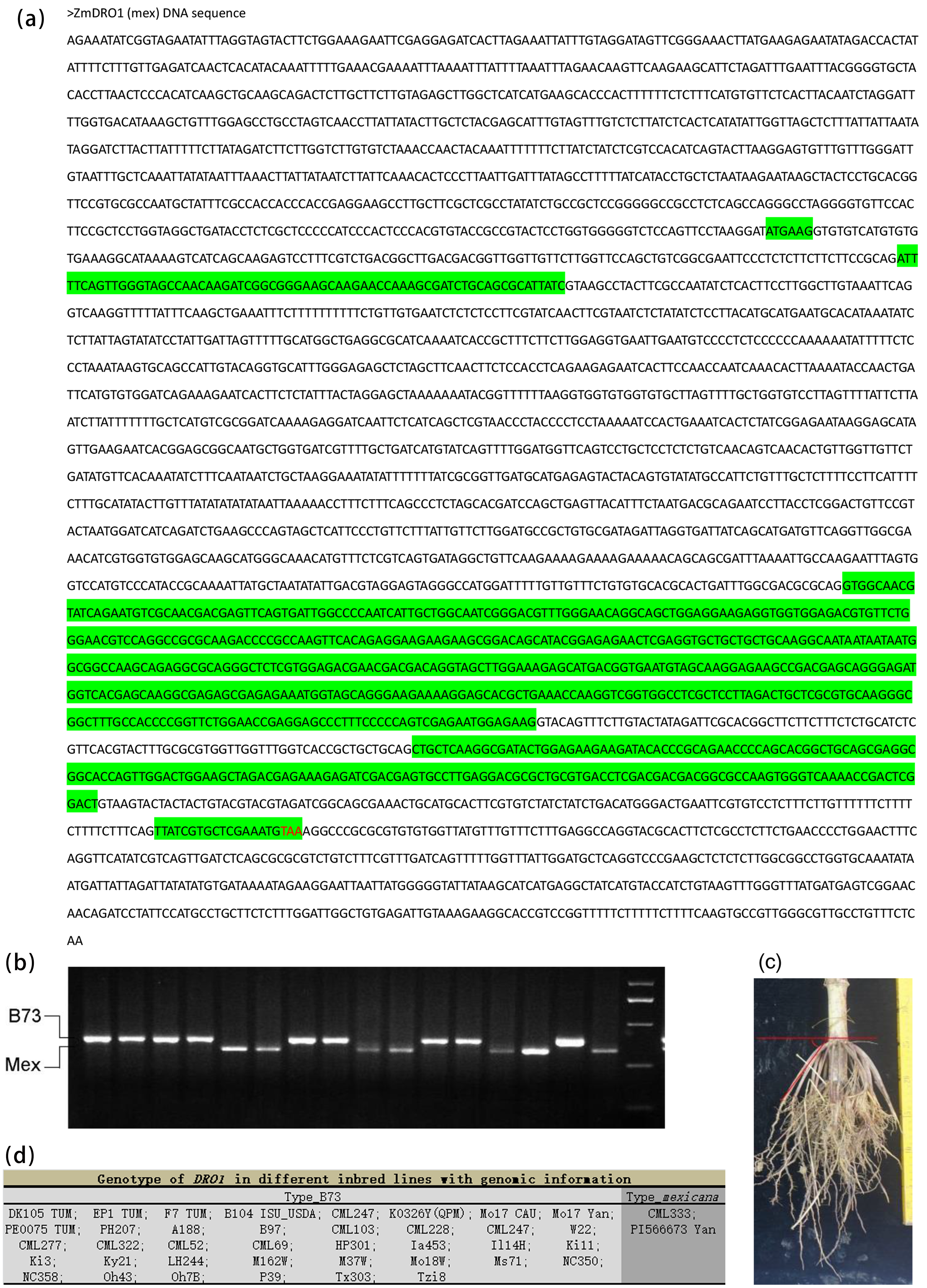

Supplement: Supplementary file 2 — Figure S2 Gene sequence of ZmDRO1 mex and allelic profile of ZmDRO1 in RILs and inbred lines with reference genomic information. [file PBI-20-2077-s005.tif]

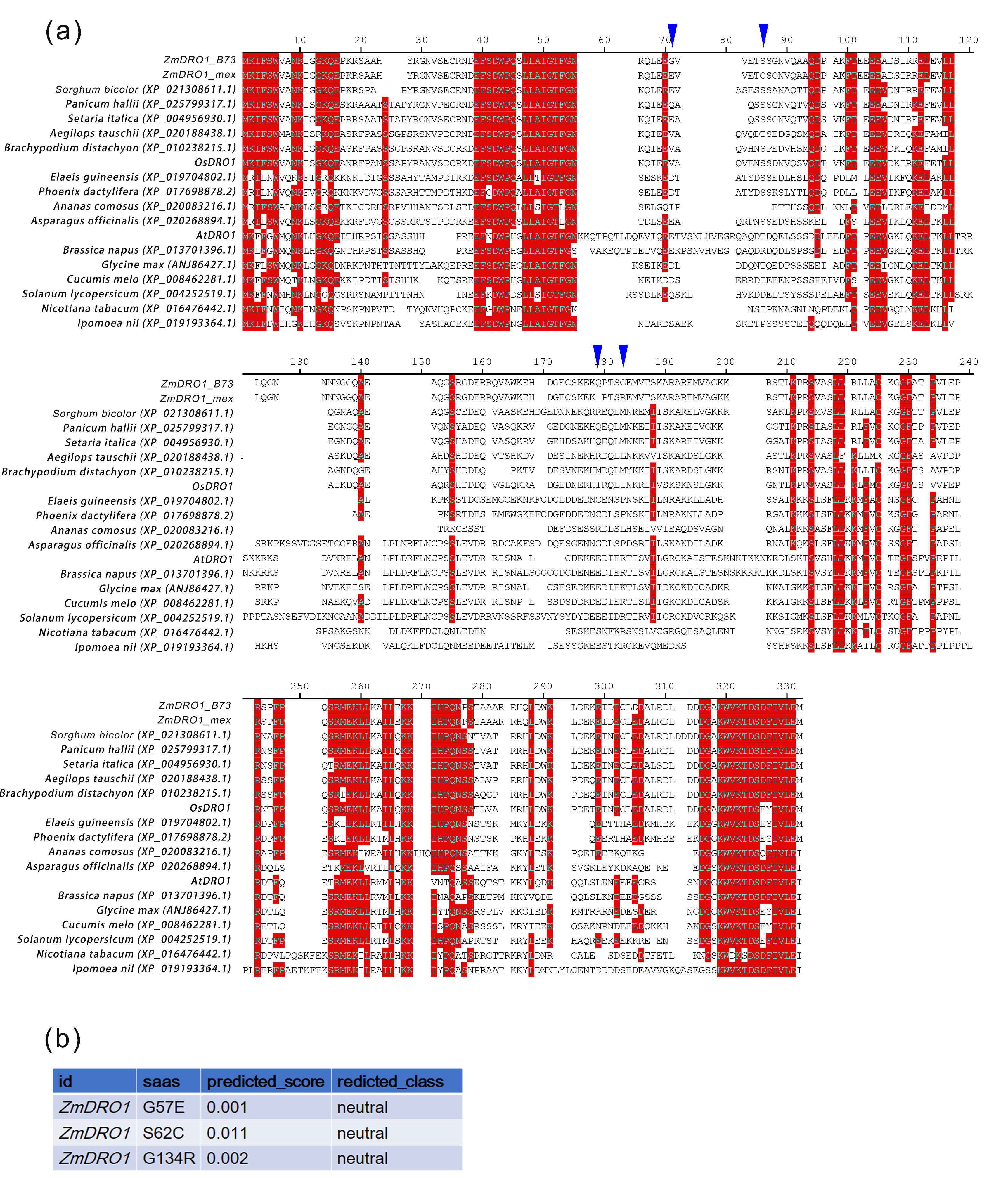

Supplement: Supplementary file 3 — Figure S3 Amino acid sequences of ZmDRO1B73 and ZmDRO1mex are highly similar. [file PBI-20-2077-s001.tif]

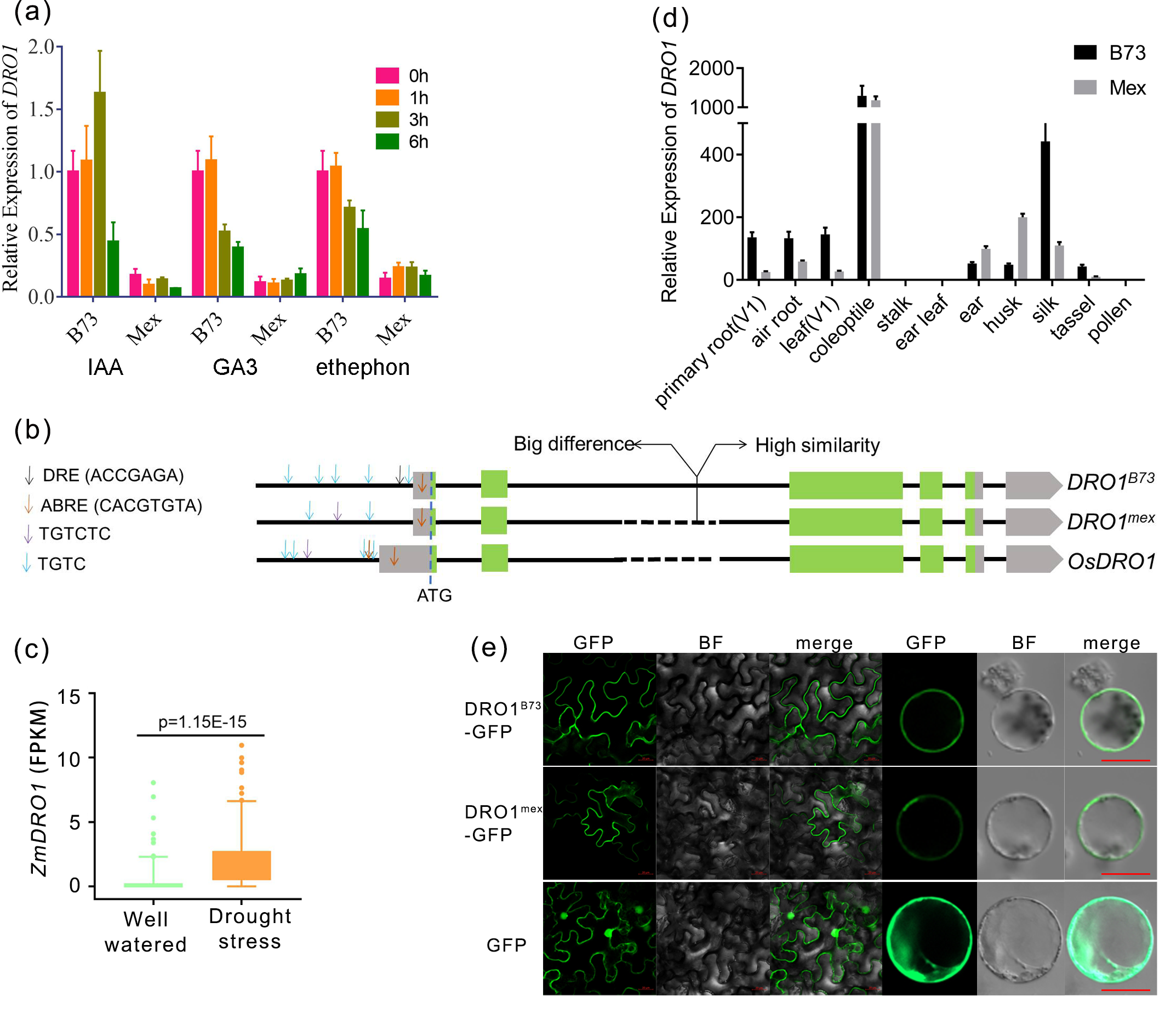

Supplement: Supplementary file 4 — Figure S4 Expression pattern and subcellular localization of ZmDRO1B73 and ZmDRO1mex. [file PBI-20-2077-s003.tif]

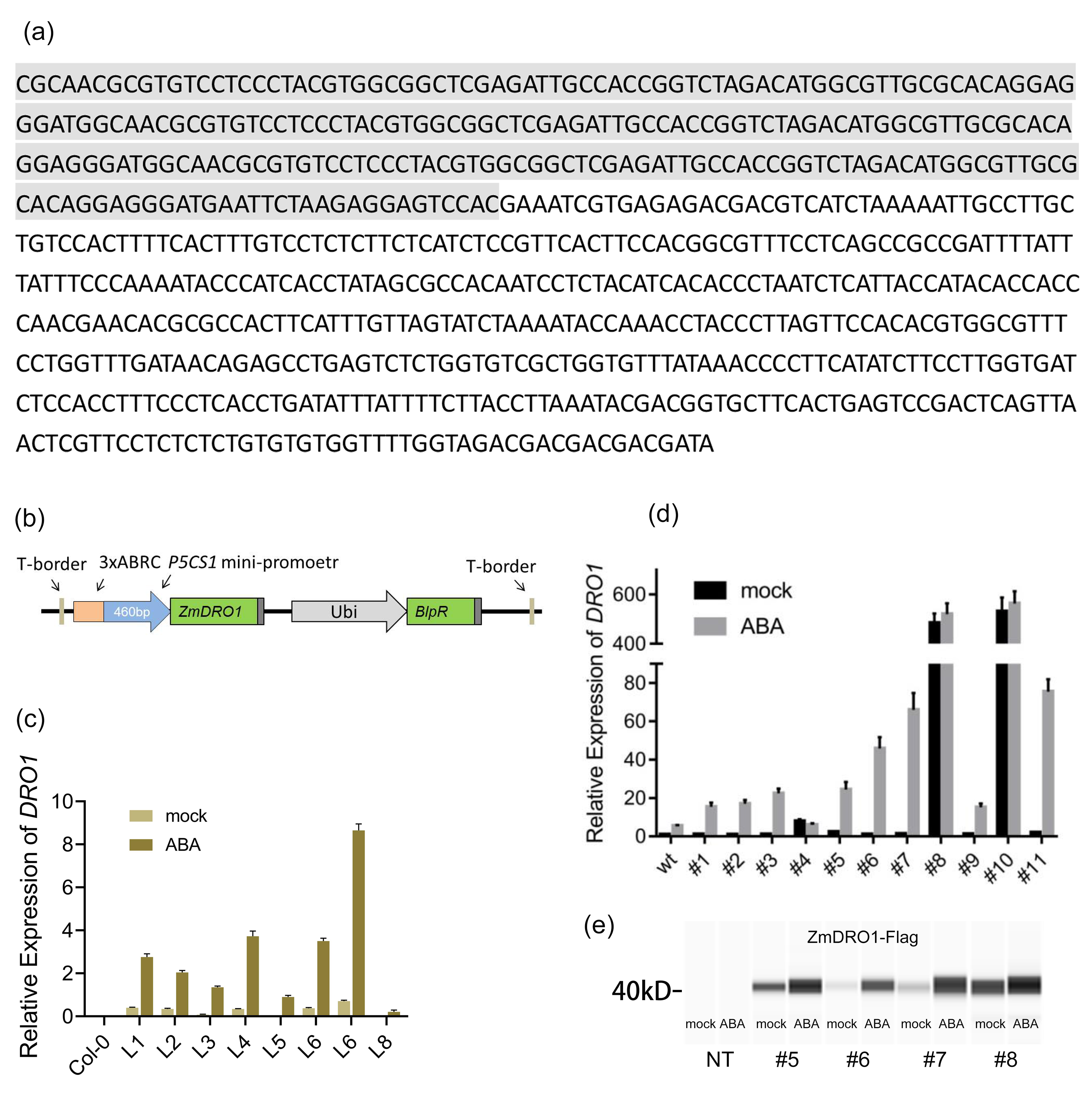

Supplement: Supplementary file 5 — Figure S5 Construction of ABA‐inducible expression cassette and the expression of various transgenic plants. [file PBI-20-2077-s007.tif]

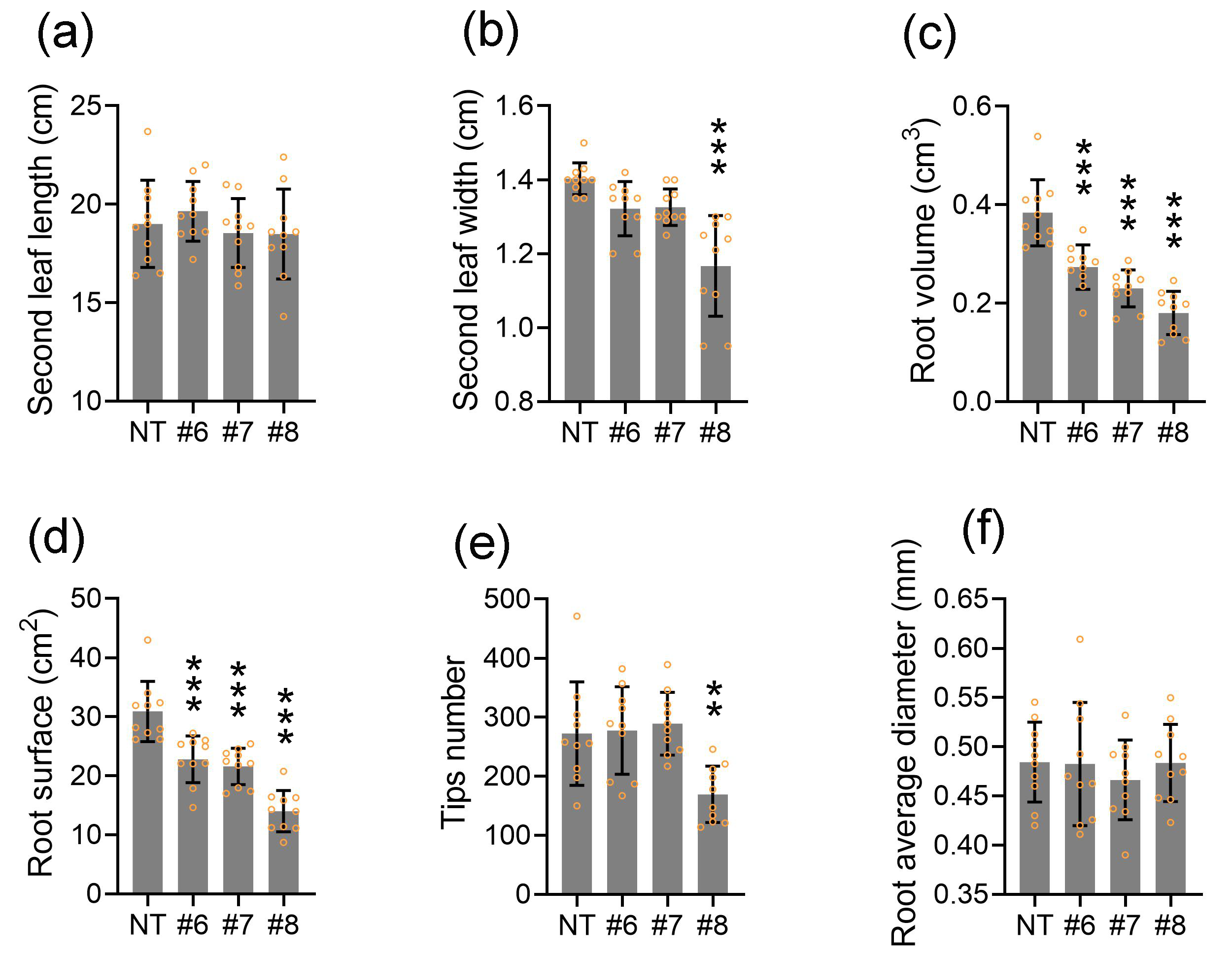

Supplement: Supplementary file 6 — Figure S6 Shoot and root phenotype of maize seedlings grown in rolled‐up germinating test paper in nutrient solution. [file PBI-20-2077-s004.tif]

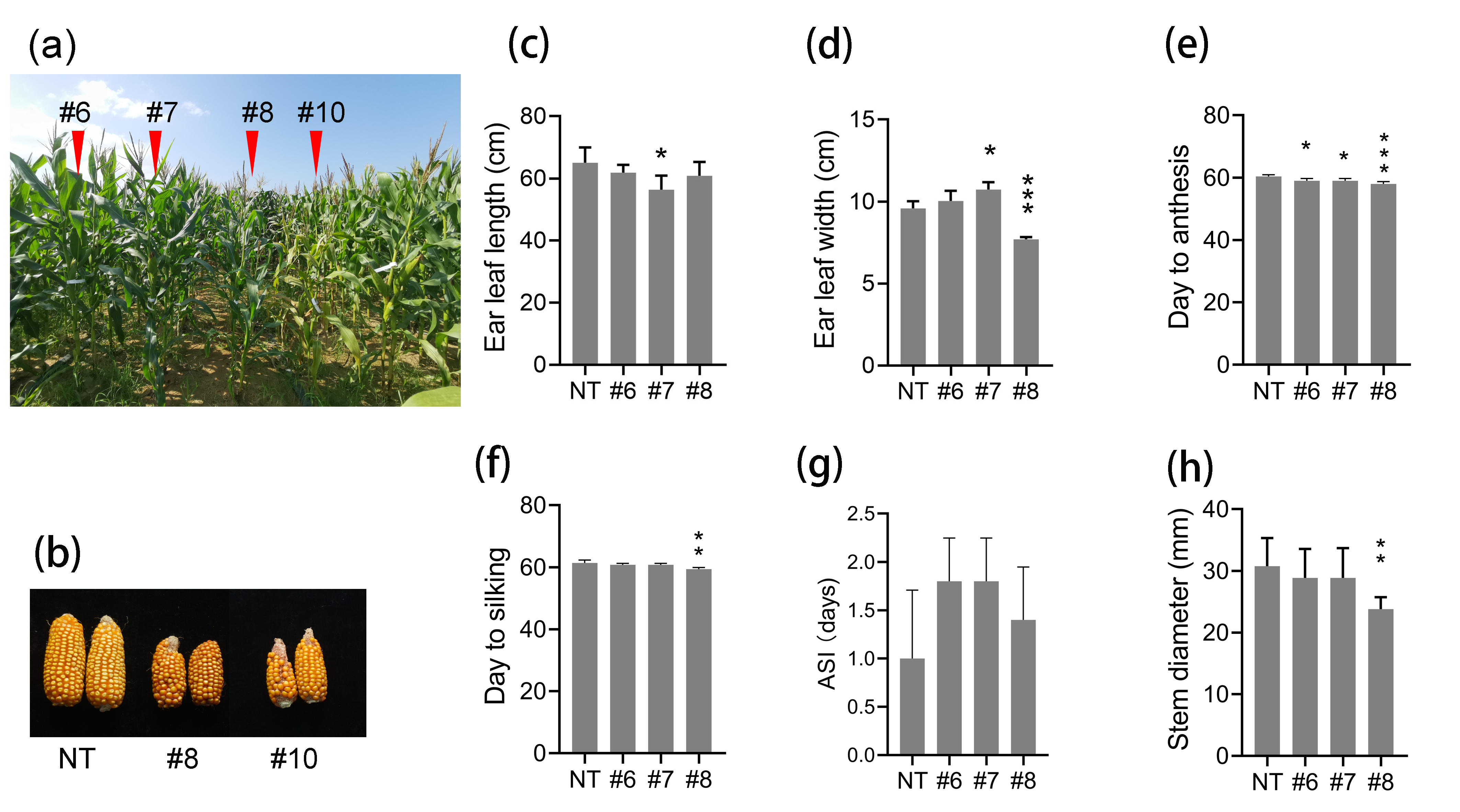

Supplement: Supplementary file 7 — Figure S7 Constitutive high expression of ZmDRO1 will severely repress plant growth. [file PBI-20-2077-s002.tif]
